# Recent advances in metacommunities and meta-ecosystem theories

**DOI:** 10.12688/f1000research.10758.1

**Published:** 2017-05-02

**Authors:** Frédéric Guichard

**Affiliations:** 1 Department of Biology, McGill University, Montreal, Quebec, Canada

**Keywords:** metacommunities, ecosystem theories, conservation

## Abstract

Metacommunity theory has provided many insights into the general problem of local versus regional control of species diversity and relative abundance. The metacommunity framework has been extended from competitive interactions to whole food webs that can be described as spatial networks of interaction networks. Trophic metacommunity theory greatly contributed to resolving the community complexity-stability debate by predicting its dependence on the regional spatial context. The meta-ecosystem framework has since been suggested as a useful simplification of complex ecosystems to apply this spatial context to spatial flows of both individuals and matter. Reviewing the recent literature on metacommunity and meta-ecosystem theories suggests the importance of unifying theories of interaction strength into a meta-ecosystem framework that captures how the strength of spatial, species, and ecosystem fluxes are distributed across location and trophic levels. Such integration predicts important feedback between local and regional processes that drive the assembly of species, the stability of community, and the emergence of ecosystem functions, from limited spatial fluxes of individuals and (in)organic matter. These predictions are often mediated by the maintenance of environmental or endogenous fluctuations from local to regional scales that create important challenges and opportunities for the validation of metacommunity and meta-ecosystem theories and their application to conservation.

## Introduction

Natural ecosystems display patterns of variation in the diversity and abundance of species over a broad range of scales. One pragmatic approach to understanding these patterns contrasts local mechanisms regulating the assembly and growth of species to regional processes of immigration and speciation ^1^. Identifying and inferring the relative importance of local and regional processes for predicting the number and relative abundance of species, as well as productivity and stability of local communities, have become defining goals of community ecology. Extending the concept of metapopulations to metacommunities ^2^, defined as a set of discrete communities partially connected through movement of individuals, has greatly contributed to the study of local versus regional control. It provided a spatially discrete framework to the broader problem of scale ^3^. Metacommunity theory also allowed studying regional processes as the collective property of local communities rather than as an external control ^4, 5^. In that respect, it contributed to understanding reciprocal rather than strictly hierarchical effects linking local and regional patterns and processes ^6^.

Theories of local versus regional control of spatially structured communities have been developed independently from the metacommunity framework ^7^. However, metacommunity theory has contributed not only a rich set of models but also experiments predicting regional patterns of intra-guild diversity, of food-web structure, and of their relationship to community stability and productivity ^8^. It is also through the metacommunity framework that theories of local and regional control have included important progress in food-web theory, phylogenetics, and spatial dynamics, including spatially explicit, individual-based, and stochastic ^9, 10^ models. Finally, the metacommunity framework has inspired a similar spatially discrete approach to the study of cycling of matter and energy at local and regional scales. The relative importance of local cycling of matter and of regional subsidies has led to the development of meta-ecosystem theories, integrating spatial flows of individuals and of (in)organic matter over regional scales and integrating species interactions and the recycling of matter within local ecosystems.

By reviewing recent progress, I will emphasize the importance of temporal fluctuations, both environmental and endogenous, and whole-ecosystem dynamics. I will also argue that such integration would be most successful within the broader theoretical framework of meta-ecosystems, where both individuals and (in)organic matter contribute to linking local and regional processes. Recent studies suggest that addressing these challenges is a key step toward resolving the reciprocal control of local and regional dynamics in natural ecosystems.

## Competitive and trophic metacommunities

### Communities result from the balance between sorting and movement

One of the main predictions of metacommunity theory is the regional distribution of species diversity based on both local competition and regional movement of species. In competitive metacommunity models, sorting, or environmental filtering, is the local process that operates through competitive exclusion, and sorting can lead to regional patterns of diversity through spatial heterogeneity in the environment. Movement among local communities is the regional process that redistributes, and eventually homogenizes, local communities, working against local sorting. Sorting is purely local in the absence of movement, whereas strong movement leads to regional sorting that favors the best competitor in the average habitat ^11^. In spatially implicit models assuming a homogeneous (all-to-all) connectivity network, intermediate movement reveals the reciprocal effects of local and regional processes: both regional and local diversity and relative species abundance then result from the balance between local sorting and immigration from the regional metacommunity. This balance can, for example, predict the contributions of both local species richness (alpha diversity) and regional species turnover (beta diversity) to community stability, through local compensatory effects, and to the maintenance of regional asynchrony in species composition ^12^.

The balance between local and regional processes in heterogeneous metacommunities was used to propose four mechanisms controlling biodiversity in competitive metacommunities ^3^ based on the relative importance of species movement and habitat heterogeneity as driving processes (species sorting, patch dynamics, mass effect, and neutral). The proposed mechanisms implicitly assumed demographic stochasticity, which stimulated the integration of a metacommunity framework to the neutral-versus-niche debate. Demographic stochasticity was treated explicitly as drift, affecting community dynamics along with competition, dispersal, and speciation ^13, 14^. Recent studies have provided a coherent framework reconciling competition and metacommunity theories ^15^, whereas others have refined statistical methods for the inference of neutral- and niche-based mechanisms of species diversity ^16^. Another important integration has recently happened with theories of biodiversity-ecosystem functions, tying into a long-standing empirical debate about the role of species richness for productivity and stability ^17^. Within that debate, the competitive metacommunity framework can reconcile apparent contradictions in observed biodiversity-ecosystem function relationships. Dispersal limitation can, for example, either prevent or saturate local sorting processes and affect the strength of species complementarity underlying the stability and productivity of local communities ^8^. Metacommunity dynamics can also resolve the combined and sometimes opposite effects of species diversity on multiple ecosystem functions ^18^.

Spatially explicit metacommunities have blurred the distinction between local and regional scales because spatiotemporal heterogeneity in the distribution of species can emerge at multiple scales rather than being *a priori*-defined. Also, when the local scale approaches the individual level, local and regional (meta)communities become arbitrarily defined along a continuum, based on assumptions about the relevant scales of processes at both ends of the continuum: competitive exclusion in local communities versus speciation and immigration over the metacommunity ^19^. This is illustrated by the application of the metacommunity framework to neutral theory. In early neutral models, the metacommunity was independent from the local community, ignoring feedbacks from local to regional levels ^20, 21^. Later studies integrated both scales through spatially explicit models where metacommunity processes emerge from the collective behavior of connected local communities ^22^. More recently, this scale continuum has been used to improve the testability of neutral predictions using patterns of spatial autocorrelation ^23^.

The competitive metacommunity framework emphasizes spatial heterogeneity among communities and as the regional mechanism of coexistence. It has also integrated the role of temporal environmental variability as a mechanism of coexistence ^24, 25^. However, the emphasis on sorting through competitive interactions has limited the understanding of other traits and interactions, and of their complexity, as drivers of species diversity and function. The study of trophic interactions allowed researchers to extend this understanding beyond the effects of competitive exclusion to networks of species interactions that are more compatible with the complexity of natural systems.

### Trophic metacommunities: networks of networks

The extension of competitive metacommunity theories to trophic metacommunities leads to a ‘spatial network of interaction networks’ ^26^. One major implication of trophic metacommunities is the generalization of species traits involved in the local and regional control of community structure and dynamics. This generalization has recently led to a deeper contribution of metacommunities to the community complexity-stability relationship, involving the integration of evolutionary and non-equilibrium dynamics to the framework.

**Figure 1.**
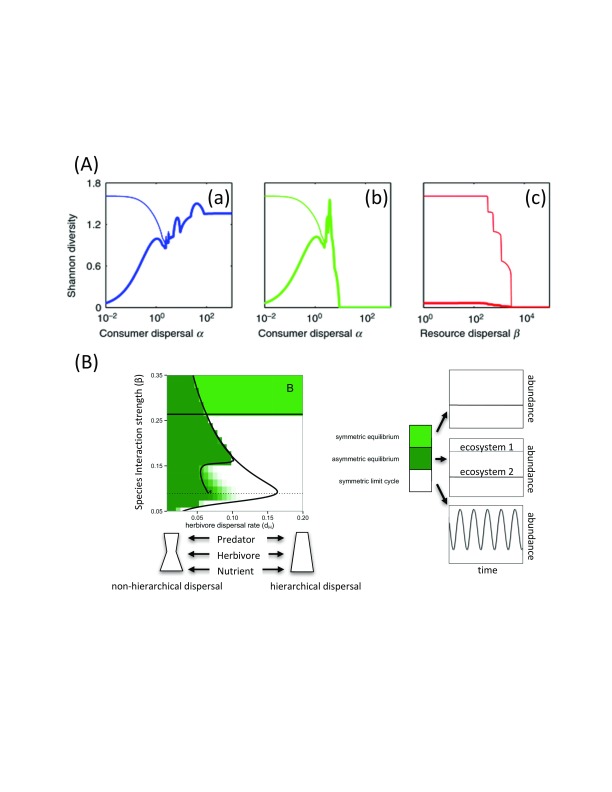
Role of spatial interaction strength across trophic levels for dispersal-diversity relationships and for regional stability. ( **A**) Diversity–dispersal relationships. (a) Consumer dispersal α varies while keeping resource dispersal β small. (b) Consumer dispersal α and resource dispersal β vary simultaneously. (c) Resource dispersal β varies while keeping consumer dispersal α small. Thick line: local diversity; thin line: regional diversity. Reprinted with permission from John Wiley & Sons, Inc. ^30^. ( **B**) Changes in qualitative dynamics in a two-patch tri-trophic meta-ecosystem from varying resource intra-specific interaction strength (β) and dispersal rates, holding all other parameters constant. At intermediate levels of β, making dispersal more non-hierarchical by decreasing middle-trophic-level dispersal rates ( *d*_H_) leads to increased stability because of the emergence of stable asymmetric equilibria. Reprinted with permission from the University of Chicago Press Books ^31^.

Trophic interactions have expanded the exploration of trait differentiation at both local and regional scales: body size ^27^, trait-mediated and density-dependent movement ^28^, or covariance between trophic position and movement ^29– 31^. It has recently led researchers to revisit the hump-shaped relationship between dispersal and diversity in competitive metacommunities. When multiple trophic levels with heterogeneous dispersal rates are considered, this expected balance between immigration and sorting can instead give rise to monotonic increase in diversity with dispersal ^30^ ( Figure 1A). The dependence of movement across trophic levels also provides a trophic context mediating local (risk-based movement) and regional (perceived habitat suitability) drivers of species diversity ^32^. Similarly, density-dependent movement in heterogeneous tri-trophic metacommunities was shown to best capture patterns of beta diversity and, more specifically, the decrease in similarity with distance observed in natural systems ^28^.

Food-web metacommunities have explored the assembly of large networks of networks as steady-state structures ^33, 34^ that drive their own stability ^35^ and productivity. This ‘network of networks’ approach provides a useful framework to integrate dispersal limitation, habitat heterogeneity, and food-web topology ^26, 36, 37^ to the complexity-stability debate initiated by May ^38^. For example, May’s predicted limit to the complexity of stable food webs could be relaxed by increasing habitat heterogeneity across metacommunities characterized by intermediate dispersal ^26, 37^. Moreover, Pillai *et al*. ^36^ showed how the interaction between dispersal and species interactions would predict the stabilizing effect of omnivorous and generalist species, which adds to predictions from non-spatial food-web theories about the stabilizing role of generalist species with multiple weak interactions ^29^.

Trophic interactions can display endogenous fluctuations and force us to reconsider the equilibrium nature of the local-regional control of food webs. In large-model food webs, Plitzko and Drossel ^35^ found that intermediate dispersal among food webs limited the occurrence of equilibrium communities but maximized the persistence of species through oscillations and dynamic coexistence. Trophic metacommunities of small food-web modules have explored the local-regional implications of spatially explicit and of oscillatory dynamics, focusing on the maintenance of regional heterogeneity from the interplay between local oscillations and movement. This approach has a long history in both experimental ^39^ and modelling ^6, 7^ studies. The use of synchrony across species and habitats has recently emerged as a powerful tool to understand and predict the dynamics and persistence of spatially extended simple food-web modules ^40, 41^. Gouhier *et al*. ^40^ showed that dispersal and correlated environmental fluctuations interact to affect metacommunity stability through their impact on both interspecific (compensatory dynamics) and intra-specific (spatial) synchrony. Using a similar approach, Pedersen *et al*. ^31^ considered heterogeneous dispersal across trophic levels and showed that tri-trophic food webs are stabilized by the formation of patterns in the presence of both weak and strong dispersal across trophic levels ( Figure 1B). These results echo the importance of weak and strong trophic species interactions for food-web stability ^42^ and suggest the possibility for an integrated treatment of spatial and species interactions ^26^.

Both competitive and trophic frameworks are readily applicable to evolutionary ecology where both sorting and selection interact with dispersal to drive the assembly of species traits in relation to their environment ^14, 43^ and where body size can be used as a proxy for trophic position ^19, 44, 45^. Moreover, phylogenetic approaches to metacommunities can identify the contribution of biogeographical history to both local and regional distribution of species ^46^, can more specifically predict increasing phylogenetic structure with community size and isolation ^47^, and have improved the testability of metacommunity predictions of patterns of beta diversity ^48^. Phylogenetic analysis of metacommunities also suggested that non-equilibrium neutral dynamics of total abundance are more compatible with observed phylogenies than steady-state communities ^49^. However, the ‘network of networks’ approach of trophic metacommunities assumes a completely open food-web network, where organic matter is lost from the metacommunity and where inorganic nutrients flow through each local habitat independently. Yet the cycling of (in)organic matter is key to understanding food-web stability ^50^ and essential to extending local-regional theories to a broad range of ecosystem functions. This understanding is a long-standing goal of ecosystem ecology and more recently has been addressed through meta-ecosystem theory.

## Meta-ecosystems: from the local cycling to regional fluxes of matter

**Figure 2.**
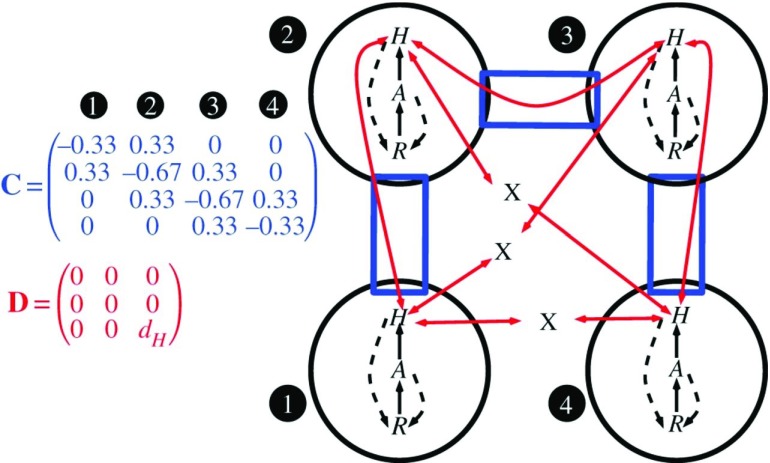
From metacommunities to meta-ecosystems: cycling of matter across spatial networks of interaction networks. Conceptual diagram for a general meta-ecosystem model that can predict the role of interaction strength between species and between locations for the exchange of individuals and matter. Local ecosystems (circles) have internal dynamics based on trophic (solid arrows) and non-trophic (dashed arrows) interactions between ecosystem compartments, which in this case are a limiting nutrient (R), autotrophs (A) and herbivores (H). Local ecosystems form a meta-ecosystem through the movement of materials and organisms, which is determined by the connectivity matrix C and the movement matrix D. The connectivity matrix indicates how the ecosystems are connected to one another (rectangular boxes), whereas the movement matrix gives the movement rates of each ecosystem compartment (two-headed arrows). Without a connection specified by the connectivity matrix, materials and organisms cannot move between ecosystems (capital X). Reprinted with permission from Elsevier ^64^.

When both spatial and interaction networks are partially closed to flows of matter, metacommunities are better described as meta-ecosystems ( Figure 2). Partially closing the cycling of matter results in its limited spatial movement across habitats in addition to its transfer across food-web compartments, including recycling. The concept of the meta-ecosystem has been introduced as an extension of metacommunity theory ^51, 52^ and has been further developed in recent years.

Spatial ecosystem dynamics is not a new concept and has been studied as part of ecosystem ecology through flows of matter across macro-habitats and through large-scale quantitative ecosystem models ^53^. However, the meta-ecosystem concept has emerged as a more specific integration of trophic metacommunities and landscape ecosystem ecology ^52^. It is partly based on the extension of cross-habitat subsidies developed in landscape ecology ^54– 56^ to reciprocal subsidies ^57, 58^. As such, meta-ecosystem theory integrates the cycling of matter across scales and can directly predict local versus regional control of community structure and ecosystem functions through the interplay between local recycling and regional fluxes of matter and their implications for the emergence of both local and regional community stability and productivity. For instance, explicit ecosystem dynamics have profound impacts on colonization-extinction dynamics in metapopulation and metacommunity models. As mentioned above, metacommunity theory predicts the local sorting of species in suitable habitats and their transient occupancy of unsuitable habitats through migration. In meta-ecosystem models, spatial fluxes and cycling of matter can become the driver of habitat suitability and result in dynamic habitat properties that are affected by both species interactions and ecosystem processes ^59, 60^. The dynamics of habitats become coupled to that of species over both local and regional scales and can lead to the emergence of facilitative interactions between competitors ^59^. These facilitative interactions result from local cycling and regional fluxes and predict cascades of extinctions following habitat destruction ^60^.

Recent studies have emphasized the importance of temporal variability in reciprocal subsidies of matter and the importance of the resulting non-equilibrium dynamics of meta-ecosystems. In simpler cases, this variability can be caused by external perturbations resulting in the release of matter associated with mortality. Harvey *et al*. ^61^ showed in experiments and in models that increasing the frequency of such perturbations leads to a transient increase in subsidies but eventually to the collapse of recipient populations that are unable to recover fast enough. Feedbacks between trophic interactions and recycling also force us to reconsider the steady-state nature of ecosystem functions. Recent studies of non-equilibrium meta-ecosystems point to the importance of recycling and spatial flows of matter for the maintenance of endogenous spatiotemporal heterogeneity that predict both strong local fluctuations and regional stability. Meta-ecosystem models where only nutrients are flowing between local ecosystems lead to the destabilization of local consumer-resource dynamics ^62^. Spatial fluxes of matter drive this destabilization, but recycling can facilitate the transition from equilibrium to cyclic dynamics. In simple two-patch meta-ecosystems, phase-locked cycles emerge from the positive feedback between recycling and passive movement of nutrients: more movement into a local ecosystem is correlated with increasing growth of the primary producer, thus destabilizing primary production. This prediction can be generalized to larger (many patch) meta-ecosystems ^63^ where the top-down control of biomass storage into inorganic form can stabilize a meta-ecosystem in the face of nutrient enrichment. For more complex finite and irregular topologies, the eigenvalues of the connectivity matrix, rather than more common topological metrics, predict the minimum movement of nutrients or of individuals that can destabilize the meta-ecosystem ^64^. Applying the meta-ecosystem framework to non-equilibrium dynamics has further revealed the relevance of endogenous spatiotemporal patterns of matter for the local versus regional control of ecosystem functions such as nutrient limitation: differences in movement rates between nutrients and other compartments lead to the emergence of nutrient co-limitation in growth over regional scales despite the lack of local mechanisms of nutrient co-limitation ^65^. These recent studies illustrate how novel metrics of landscape heterogeneity, combined with a knowledge of spatial and species interaction strength, can lead to predictions that are tailored to specific empirical study systems. Finally, non-equilibrium meta-ecosystem theories are also relevant to conservation endeavors by predicting how the emergence of multiple scales of spatiotemporal patterns of abundance can inform decisions on the optimal size and spacing of local protected areas within regional reserve networks ^66, 67^.

## Conclusions

Metacommunity theory has broadened its range of predictions by integrating trophic interactions, spatially explicit movement, evolutionary processes, and phylogenetic history. By giving rise to meta-ecosystem theories through the integration of recycling and movement of matter, it has further emphasized the interactions between local and regional scales for the regulation of community structure and ecosystem functions. Overall, recent metacommunity and meta-ecosystem theories have benefited from the simplified representation of ecosystems as ‘networks of networks’. This approach has stressed the importance of differential movement rates across ecosystem compartments for predicting regional ecosystem stability. It more generally calls for the integration, both conceptually and methodologically, of spatial and trophic interaction strength. One fundamental challenge that materialized from these efforts is the potential for non-equilibrium dynamics and emerging spatiotemporal patterns that can drastically affect both local and regional community structure and ecosystem functions. Indeed, metacommunity and meta-ecosystem theories predict when local fluctuations, far from averaging out over regional scales, propagate and cause variability in these properties across spatial and temporal scales. This is a fundamental challenge for the development of meta-ecosystem theories and for testing their predictions using experimental metacommunity approaches that are based on steady states and that ignore the recycling and spatial fluxes of matter ^68^. This challenge could constitute an important hurdle to reaching regional conservation goals.
